# Supplementary material for: The efficacy of preoperative evolocumab-rosuvastatin combination therapy in patients with ST-elevation myocardial infarction
Source: PLoS One. 2026 Jan 2;21(1):e0339501. doi: 10.1371/journal.pone.0339501 (PMC12758709; doi:10.1371/journal.pone.0339501)
Supplement: S1 File — (DOCX) [file pone.0339501.s001.docx]

| Section/Topic | Item | Checklist Item | Reported | Page |
| --- | --- | --- | --- | --- |
| Title & Abstract | 1a | Identification as RCT in title | ✓ | 1 |
|  | 1b \| | Structured summary with design, methods, results, conclusions | ✓ | 1 |
| Introduction | 2a | Scientific background and rationale | ✓ | 2-3 |
|  | 2b | Specific objectives/hypotheses | ✓ | 3 |
| Methods | 3a | Description of trial design | ✓ | 3 |
|  | 3b | Important changes to methods after commencement | N/A |  |
| Participants | 4a | Eligibility criteria | ✓ | 3 |
|  | 4b | Settings/locations of data collection | ✓ | 3 |
| Interventions | 5 | Interventions for each group | ✓ | 4 |
| Outcomes | 6a | Primary/secondary outcomes clearly defined | ✓ | 4 |
|  | 6b | Changes to outcomes after commencement | N/A |  |
| Sample Size | 7a | How sample size was determined | ✓ | 4 |
|  | 7b | Interim analyses/stopping rules | N/A |  |
| Randomization | 8a | Method of random sequence generation | ✓ | 4 |
|  | 8b | Type of randomization (simple) | ✓ | 4 |
|  | 9 | Allocation concealment mechanism | ✓ | 4 |
|  | 10 | Implementation of random sequence \| | ✓ (research coordinator) \| | 4 |
| Blinding | 11a | Who was blinded (outcome assessors) | ✓ | 5 |
|  | 11b | Similarity of interventions | N/A (open-label) |  |
| Statistical Methods | 12a | Statistical methods for primary outcomes | ✓ | 6 |
|  | 12b | Methods for additional analyses | N/A |  |
| Results | 13a | Flow of participants (diagram) | ✓(Fig 1) | 5 |
|  | 13b | For each group, losses and exclusions after randomisation, together with reasons | ✓(Fig 1) | 5 |
|  | 14a | Dates of recruitment/follow-up | ✓ | 3 |
|  | 15 | Baseline demographic/clinical data | ✓ (Table 1) | 5 |
|  | 16 | each group, number of participants (denominator) included in each analysis and whether the analysis was by original assigned groups | ✓ (n=40 each) | Results |
|  | 17a | Outcomes per group + effect estimates | ✓ | 5-6 |
|  | 17b | Binary outcomes with absolute effects | ✓ | 5-6 |
| Harms | 19 | All important harms or unintended effects in each group | ✓ | 6 |
| Discussion |  |  |  |  |
| Limitations | 20 | Trial limitations, addressing sources of potential bias, imprecision, and, if relevant, multiplicity of analyses | ✓ | 7 |
|  | 21 | Generalisability (external validity, applicability) of the trial findings | ✓ | 7 |
|  | 22 | Interpretation consistent with results, balancing benefits and harms, and considering other relevant evidence | ✓ | 7 |
| Other Information | 23 | Registration number/registry | ✓ (ChiCTR2500099498) | 3 |
|  | 24 | Protocol access statement | ✓ (available on request) | Data Avail |
|  | 25 | Funding sources | ✓ (Liaocheng Key Research and Development Plan) | Funding |

CONSORT 2010 checklist
