# Supplementary material for: The efficacy of preoperative evolocumab-rosuvastatin combination therapy in patients with ST-elevation myocardial infarction
Source: PLoS One. 2026 Jan 2;21(1):e0339501. doi: 10.1371/journal.pone.0339501 (PMC12758709; doi:10.1371/journal.pone.0339501)
Supplement: Attachment — Submitted filename: Response to Reviewers.docx [file pone.0339501.s004.docx]

PONE-D-25-32807

The efficacy of preoperative evolocumab-rosuvastatin combination therapy in patients with ST-elevation myocardial infarction

Dear Editor and Reviewers,

We sincerely appreciate the time and effort dedicated to reviewing our manuscript and providing constructive feedback. We have carefully addressed each comment to improve the quality and clarity of our work. Below, we provide a point-by-point response to the editor reviewers’ comments, with corresponding revisions highlighted in the revised manuscript (tracked changes file).

Response to Editor :

1. deposit laboratory protocols in protocols.io:

It's a good suggestion to deposit laboratory protocols in protocols.we have deposited our laboratory protocols in protocols.io to enhance the reproducibility of results.

DOI: dx.doi.org/10.17504/protocols.io.n2bvje6mwgk5/v1

(Private link for reviewers:https://www.protocols.io/private/107F70C8702E11F08A690A58A9FEAC02 to be removed before publication.)

2. meets PLOS ONE's style requirements：

We have tried to ensure that our manuscripts meet PLOS ONE's style requirements, including document naming requirements and modified accordingly. However, due to our limited ability, please assist in correcting any inadequacies, thank you again for your efforts.

3. data will be submitted to a repository:

Thanks for the reminder, We have now uploaded our complete minimal dataset to Figshare, a PLOS-approved repository:

· Repository: Figshare

· DOI: 10.6084/m9.figshare.29986402

4. Clinical Trial registered and provide the complete date range for participant recruitment and follow-up

We have stated the name of the registry and the registration number in the submission data and on the title page of the manuscript and the reasons for not registering this study before enrolment of participants started in the Methods section. we confirm that all related trials are registered by stating: The authors confirm that all ongoing and related trials for this drug/intervention are registered.

.The trial was registered at the Chinese Clinical Trial Registry (ChiCTR2500099498).

This trial was not pre-registered due to initial failure to fully recognize the importance of early registration and lack of familiarity with the registration process. However, a retrospective registration was conducted at the Chinese Clinical Trials Registry (ChiCTR2500099498) before data was analysised, and all data were ensured complete, transparent, and non-selective reporting.The authors confirm that all ongoing and related trials for this drug/intervention are registered.

We have provided the complete date range for participant recruitment and follow-up in the methods section. The dates of start and end of the recruitment period for this study are July 23, 2023 and April 22, 2024, respectively. The last follow-up date was October 26, 2024.

5. amend manuscript to include your abstract after the title page：

Thank you for the reminder, we have done as you reminded.

6. include a caption for figure 1：

Thank you for your suggestion, we have added a caption for figure 1.

Flow of participants through each stage of the randomized trial. The diagram adheres to the CONSORT 2010 statement.A total of 112 acute ST-elevation myocardial infarction (STEMI) patients were screened between July 2023 and April 2024 at Liaocheng People's Hospital. After exclusions (n=32), 80 patients were randomized to intervention (n=40) or control (n=40). All participants completed the 6-month follow-up and were included in the intention-to-treat（ITT） analysis.

Ethical approval: Institutional Ethics Committee of Liaocheng People's Hospital (LCSY-2023009).

Trial registration: Chinese Clinical Trial Registry (ChiCTR2500099498).

7. the reviewer comments include a recommendation to cite specific previously published works,：

We have reviewed and evaluated the publication and determine that it is partly relevant and should be cited. “Recent trials with bempedoic acid (13) have demonstrated additional LDL-C lowering options for statin-intolerant patients. While our study focused on PCSK9 inhibition, the potential synergistic effects of combining bempedoic acid with evolocumab warrants investigation, particularly given their complementary mechanisms targeting cholesterol biosynthesis and clearance respectively. Future studies should explore whether triple therapy (statin+bempedoic acid+PCSK9 inhibitor) could provide incremental benefits in acute coronary syndromes."

Response to Reviewer 1:

1. Baseline LDL Cholesterol Levels:

Thank you for raising this important point. Due to the acute onset of acute myocardial infarction, the patient's diet was uncertain at the time of visit, and the emergency examination items in our hospital did not include LDL cholesterol, and LDL-C testing was not performed before starting statin and elolumab treatment. Considering that the grouping was randomized and there was no significant difference in basic data between the two groups, it is speculated that there may be no significant difference in baseline LDL-C between the two groups, and the difference in LDL-C after treatment is mainly related to treatment. Of course, if there is baseline data, it may be more illustrative, and we will take this into account in future studies.

2. Markers of Cardiac Injury (CK/CKMB) and Inflammation

We agree that additional biomarkers could provide further insights. TNI instead of CK and CK-MB levels was measured during the study, and no significant differences were observed between the groups，CRP was also assessed as a more accessible inflammatory marker, and no significant differences were observed between the groups (data now included in Table 6).

Table6: CRPand TNI Changes on Day 1

|  | control group | treatment group | z | p |
| --- | --- | --- | --- | --- |
| CRP(mg/L) | 7.52（3.24～20.10） | 10.14（3.01～17.69） | -0.178 | 0.859 |
| TNI(ng/ml) | 8.00(4.70～14.00) | 7.55(3.03～14.25) | -0.236 | 0.814 |

3. Single-Dose Evolocumab and Treatment Duration:

Thanks for the suggestion. A possible reason for the results to be less significant is because only 1 dose of evolocumab was given. If this was continued, then the LDL lowering effect should still be significant and this could translate into better outcomes for the treatment arm. This has been confirmed by the FORIER study which supports sustained treatment. On the basis of guideline-guided drug therapy, elolumab combined with statin intensive lipid-lowering and anti-inflammatory therapy was applied before emergency PCI to observe the efficacy of a single dose. This is the novelty of this study.

Response to Reviewer 2:

1. Abstract Misprint:

We apologize for the typographical errors in the abstract. We have completely rewritten the abstract to eliminate any potential misprints and improve clarity. The revised version now follows a structured format with clear Background/Methods/Results/Conclusions sections, includes all key numerical findings with precise P-values, and maintains balanced interpretation of results. The clinical trial registration number has been prominently displayed.

2. Primary Endpoint Clarification:

Thank you for this important methodological observation. We confirm that the primary endpoint (6-month MACE) was indeed the basis for our sample size calculation, as stated in the Methods section (2.5 Statistical Analysis). We have now added explicit confirmation: "Sample size was calculated based on the primary endpoint of 6-month MACE incidence, assuming 80% power to detect a 25% absolute reduction between groups (α=0.05, two-sided).

3. Angina Assessment Details:

Additional details on angina assessment (e.g., CCS classification, symptom diaries) have been included in the Methods and Results sections to improve transparency. We have expanded both Methods and Results sections regarding angina assessment:

- Methods (2.4 Outcome Measures): Added "Angina was assessed according to CCS classification criteria through scheduled clinic visits and documented using standardized case report forms."

- Results (3.2.4): Added "All reported angina episodes were physician-adjudicated and required typical symptoms of ischemia with or without objective evidence of ischemia (ECG changes or imaging evidence

4. Bempedoic Acid and Novel Therapies:

We appreciate this valuable suggestion and have substantially expanded the Discussion section to incorporate recent evidence on bempedoic acid:

- Added new paragraph: "Recent trials with bempedoic acid (PMID: 38017541) have demonstrated additional LDL-C lowering options for statin-intolerant patients. While our study focused on PCSK9 inhibition, the potential synergistic effects of combining bempedoic acid with evolocumab warrants investigation, particularly given their complementary mechanisms targeting cholesterol biosynthesis and clearance respectively. Future studies should explore whether triple therapy (statin+bempedoic acid+PCSK9 inhibitor) could provide incremental benefits in acute coronary syndromes."

Additional Revisions

1. Provide rationale for the chosen rosuvastatin dose (10 mg). We added justification in the Introduction (page 3, paragraph 2):

Medium-dose rosuvastatin (10 mg) was selected to balance efficacy and safety in Chinese populations, as higher doses may increase hepatotoxicity risk without proven incremental benefits in acute settings [4].

2. Discuss the clinical relevance of early LDL-C reduction in STEMI. Expanded in Discussion (page 9, paragraph 2):

Rapid LDL-C reduction within 24 hours may attenuate plaque inflammation and microvascular dysfunction, potentially explaining the observed STR improvement.

3.Grammar/Clarity: Polished language throughout (e.g., "postoperative day 1" → "post-PCI day 1").

4. All changes are tracked in the revised manuscript, and new references/citations have been added as appropriate.

We believe these revisions have strengthened the manuscript and addressed all critiques. Thank you for considering our work for publication in PLOS ONE.

Thank you again for your time and expertise.

Sincerely,

Zuo-lin Fu, on behalf of the authors

Email: fzl04@163.com

Attachments:

1. *Revised Manuscript with Track Changes*

2. *Manuscript (clean version)*

3. *Response to Reviewers*
