# Supplementary material for: The efficacy of preoperative evolocumab-rosuvastatin combination therapy in patients with ST-elevation myocardial infarction
Source: PLoS One. 2026 Jan 2;21(1):e0339501. doi: 10.1371/journal.pone.0339501 (PMC12758709; doi:10.1371/journal.pone.0339501)
Supplement: Attachment — Submitted filename: Response to Reviewers1.docx [file pone.0339501.s005.docx]

*PONE-D-25-32807R1*

*The efficacy of preoperative evolocumab-rosuvastatin combination therapy in patients with ST-elevation myocardial infarction*

Dear Dr. Kamal Sharma and Reviewers,

Thank you for giving us the opportunity to submit a revised version of our manuscript titled "The efficacy of preoperative evolocumab-rosuvastatin combination therapy in patients with ST-elevation myocardial infarction" (Manuscript ID: PONE-D-25-32807R1). We sincerely appreciate the editors and reviewers for your time and valuable comments, which have greatly helped us in improving the quality of our manuscript.

We have carefully addressed the points raised by the reviewers. The changes made in the manuscript are highlighted in the "Revised Manuscript with Track Changes" file. An additional clean version of the revised manuscript has also been submitted.

Response to Editor :

1. deposit laboratory protocols in protocols.io:

It's a good suggestion to deposit laboratory protocols in protocols.we have deposited our laboratory protocols in protocols.io to enhance the reproducibility of results.

DOI: dx.doi.org/10.17504/protocols.io.n2bvje6mwgk5/v1

(Private link for reviewers:https://www.protocols.io/private/107F70C8702E11F08A690A58A9FEAC02 to be removed before publication.)

2. *review reference list to ensure that it is complete and correct：*

W*e have reviewed the references in our manuscript. While the reference list is well-structured and as of November 17, 2025, no articles have been marked as “Retracted” or “Expression of Concern”， one minor error is identified and corrected in the author list in Reference 10.*

Response to Reviewer #4:

We thank the reviewer for their positive feedback and valuable suggestions to further strengthen our manuscript. We have addressed the specific comments as follows:

1. Comment: "Page 1-3, line 25-84: The introduction builds good context on CAD and PCI but lacks specific evidence linking psychological adaptation to clinical outcomes. Add a few sentences citing studies that report the poor resilience leading to worse cardiac rehabilitation adherence or higher anxiety."

· Response: We thank the reviewer for this insightful suggestion. While our study primarily focuses on the pharmacological intervention, we recognize the importance of the patient's psychological state in overall recovery. We have now added a sentence in the Introduction section to acknowledge this aspect and cite relevant literature:

"Beyond pharmacological therapy, a patient's psychological adaptation, such as low resilience, has been linked to poorer adherence to cardiac rehabilitation and higher levels of anxiety, which may indirectly influence long-term clinical outcomes [2]"

[2] *Bertolín-Boronat C, Marcos-Garcés V, Merenciano-González H, Martínez Mas ML, Climent Alberola JI, Perez N, López-Bueno L, Esteban Argente MC, Valls Reig M, Arizón Benito A, Payá Rubio A, Ríos-Navarro C, de Dios E, Gavara J, Jiménez-Navarro MF, Chorro FJ, Sanchis J, Bodi V. Depression, Anxiety, and Quality of Life in a Cardiac Rehabilitation Program Without Dedicated Mental Health Resources Post-Myocardial Infarction. J Cardiovasc Dev Dis. 2025 Mar 4;12(3):92. doi: 10.3390/jcdd12030092.*

2. Comment: "Page 18, 174-178: Conclusion is good but could be more impactful via adding statements like 'Future longitudinal studies should evaluate how interventions that strengthen coping skills can enhance resilience and reduce cardiovascular event recurrence.'"

· Response: We agree that this is an important direction for future research. We have expanded the Conclusion section to include this perspective:

"Furthermore, future longitudinal studies should explore how interventions combining intensive lipid-lowering therapy with psychosocial support, such as those aimed at strengthening coping skills and resilience, could potentially enhance overall patient outcomes and reduce cardiovascular event recurrence."

3. Comment: "Page 13, Line 66: Clear structure but lacks details on scale validation and sampling. Needs to inform the handling of missing data or incomplete questionnaire."

· Response: We thank the reviewer for pointing this out. We have added details in the Methods section (2.5 Statistical Analysis) regarding data handling:

"The analysis followed the intention-to-treat principle. For the primary and secondary endpoints, there were no missing data as all 80 randomized participants completed the 6-month follow-up. Any incomplete questionnaire items in the case report forms were clarified by direct contact with the attending physician before database locking."

4. Comment: "Proof read the overall article again, and correct any grammatical error that might be in place."

· Response: We have thoroughly proofread the entire manuscript to correct grammatical errors and improve language clarity. This has been done throughout the "Revised Manuscript with Track Changes."

Response to Reviewers #1, #2, and #3:

We thank the reviewers for their previous comments,which have been adequately addressed in the prior revision round, and for their current acceptance of the manuscript.

We believe these revisions have strengthened the manuscript and addressed all critiques. Thank you for considering our work for publication in PLOS ONE.

Thank you again for your time and expertise.

Sincerely,

Zuo-lin Fu, on behalf of the authors

Email: fzl04@163.com

Attachments:

1. *Revised Manuscript with Track Changes*

2. *Manuscript (clean version)*

3. *Response to Reviewers*
